# AtSIG6, a plastid sigma factor from Arabidopsis, reveals functional impact of cpCK2 phosphorylation

**DOI:** 10.1111/j.1365-313X.2010.04138.x

**Published:** 2010-04

**Authors:** Jennifer Schweer, Hacer Türkeri, Brigitte Link, Gerhard Link

**Affiliations:** Laboratory of Plant Cell Physiology and Molecular Biology, University of Bochum, Universitaetsstr. 15044780 Bochum, Germany

**Keywords:** plant sigma factor, plastid transcription kinase, multisite phosphorylation control, chloroplast transcription, Arabidopsis phenotype, point mutants

## Abstract

Plastids contain sigma factors, i.e. gene-regulatory proteins for promoter binding and transcription initiation. Despite the physical and functional similarity shared with their prokaryotic counterparts, the plant sigma factors have distinguishing features: most notably the existence of a variable extra sequence comprising their N-terminal portions. This distinct architecture is reflected by functional differences, including phosphorylation control by organellar protein kinase(s) closely related to nucleocytosolic, rather than bacterial-type, enzymes. In particular, cpCK2, a nuclear-coded plastid-targeted casein kinase 2, has been implicated as a key component in plant sigma factor phosphorylation and transcriptional regulation (*Eur. J. Biochem.* 269, 2002, 3329; *Planta*, 219, 2004, 298). Although this notion is based mainly on biochemical evidence and *in vitro* systems, the recent availability of Arabidopsis sigma knock-out lines for complementation by intact and mutant sigma cDNAs has opened up new strategies for the study of transcription regulatory mechanisms *in vivo*. Using Arabidopsis sigma factor 6 (AtSIG6) as a paradigm, we present data suggesting that: (i) this factor is a substrate for regulatory phosphorylation by cpCK2 both *in vitro* and *in vivo*; (ii) cpCK2 phosphorylation of SIG6 occurs at multiple sites, which can widely differ in their effect on the visual and/or molecular phenotype; (iii) *in vivo* usage of the perhaps most critical cpCK2 site defined by Ser174 requires (pre-)phosphorylation at the n + 3 serine residue Ser177, pointing to ‘pathfinder’ kinase activity capable of generating a functional cpCK2 substrate site.

## Introduction

Plant cells are tripartite genetic systems consisting of three transcriptionally active compartments, i.e. the nucleus, the mitochrondria and the plastids. The latter contain two principally different forms of RNA polymerases for transcription of a full complement of organellar genes in normal (wild-type) plants. Nucleus-encoded polymerase (NEP) is a single-subunit enzyme closely related to those of T7/T3 phages and mitochondria. In contrast, plastid-encoded polymerase (PEP) is a multi-subunit bacterial-type enzyme with α-, β- and β′-equivalent core subunits that are encoded by plastid genes (Maliga, 1998; Hess and Börner, 1999). It has become clear, however, that the core polypeptides are embedded into a much larger functional complex, made up of nucleus-encoded polypeptides, most of which seem to represent chloroplast versions of ‘eukaryotic’ nucleo/cytosolic proteins (Pfannschmidt *et al.*, 2000; Suzuki *et al.*, 2004; Pfalz *et al.*, 2006).

An intriguing feature of PEP transcription is the involvement of typical ‘prokaryotic’, yet nucleus-encoded, sigma initiation factors (for review see e.g. Gross *et al.*, 1998; Burgess and Anthony, 2001; Helmann, 2009). Plastids usually contain a small set of these factors, each of which reveals the principal sigma domains within its conserved C-terminal region (CR) (Campbell *et al.*, 2002; Murakami *et al.*, 2002). Unlike many bacterial sigma factors, however, the plant factors have a highly variable unconserved region (UCR) comprising their N-terminal portions (Shiina *et al.*, 2005; Lysenko, 2007). Although the role of UCR has long remained enigmatic, recent work suggests that it is critically involved in specifying the visual and molecular phenotype (Kubota *et al.*, 2007; Schweer *et al.*, 2009).

*Arabidopsis thaliana* contains a family of six sigma genes, named *AtSig1*–*AtSig6* (Isono *et al.*, 1997; Tanaka *et al.*, 1997; Fujiwara *et al.*, 2000; Hakimi *et al.*, 2000). Two lines of evidence have led to an assignment of the gene products as true sigma factors: (i) *in vitro* transcription and DNA-binding experiments using the bacterially expressed recombinant factors and *Escherichia coli* core RNA polymerase; and (ii) sigma knock-out and antisense plants as tools that allow us to establish causal relationships between sigma genes and phenotypic traits *in vivo* (Hanaoka *et al.*, 2003; Privat *et al.*, 2003).

Despite general agreement on the existence of ‘true’ sigma factors in plants, it has turned out to be more difficult than anticipated to assign each individual factor a well-defined role in gene (promoter)-specific transcription, with noticeable consequences for plant development and function. This is in part because of a certain level of functional overlap between factors, which helps maintain the overall transcription program, even in adverse situations (Kanamaru and Tanaka, 2004; Lysenko, 2007). Another, related, reason is that sigma factor steady-state levels do not necessarily correlate with the magnitude of downstream effects in plastid gene expression. In part, this can be assigned to reversible protein modification, such as phosphorylation, which results in altered promoter binding, and thus greater flexibility of transcription (Tiller and Link, 1993).

Available evidence (Baginsky *et al.*, 1997, 1999; Baena-Gonzalez *et al.*, 2001) suggests that a PEP-associated Ser/Thr protein kinase, termed plastid transcription kinase (PTK), is a major player in plastid sigma factor phosphorylation. Cloning, sequencing and functional characterization revealed that the catalytically active component is a nucleus-encoded and chloroplast-targeted protein closely related to the α-subunit of nucleocytosolic casein kinase 2 (CK2) (Pinna, 1990), which was hence named cpCK2 (Ogrzewalla *et al.*, 2002). Subsequent work established the presence of a single gene for cpCK2 in a number of plant species, including Arabidopsis (Loschelder *et al.*, 2004; Salinas *et al.*, 2006). Nevertheless, despite identification and characterization of the transcription kinase itself, the extent to which phosphorylation control is responsible for the activation or inactivation of plant sigma factors *in vivo* has not yet been reported.

To help clarify this question, we focused our attention on one of the Arabidopsis sigma factors, AtSIG6, for which mutant lines with readily discernible phenotypes are available (Ishizaki *et al.*, 2005; Loschelder *et al.*, 2006; Schweer *et al.*, 2006, 2009; Coll *et al.*, 2009). We asked whether AtSIG6 could serve as a PTK/cpCK2 target and, if so, which phosphorylation site(s) might be functionally relevant in Arabidopsis *in vivo* compared with the bacterially expressed recombinant protein *in vitro*. Using site-directed mutagenesis and retransformation of an *AtSig6* knock-out line, we present evidence suggesting phosphorylation control of SIG6 activity by cpCK2 and probably one other protein kinase.

## Results

### Localization and selection of putative PTK/cpCK2 phosphorylation sites on sigma factor AtSIG6 using prediction tools and sequence alignments

*In vitro* experiments with authentic chloroplast sigma factors had provided initial clues suggesting that their activity depends on phosphorylation state (Tiller and Link, 1993). In addition, recombinant sigma factor SaSIG1 from mustard was shown to be a cpCK2 substrate (Ogrzewalla *et al.*, 2002), although the functional consequences were not investigated. It thus remained to be established if (cpCK2) phosphorylation might have a regulatory effect on plant sigma factors, whether or not such a mechanism plays a role both *in vitro* and *in vivo*, and where exactly the relevant sites might be located on the substrate protein(s). To gain information whether AtSIG6 can be a potential substrate for CK2 phosphorylation, we therefore searched for putative sites in the derived protein sequence.

Consensus phosphorylation site motifs for (nucleocytosolic) CK2 often conform to the sequence motifs S*/T*xxEx and S*/T*xxDx, respectively (Pinna, 1990; Meggio and Pinna, 2003) (Table 1, bottom). In addition, as an alternative to the acidic residues aspartate or glutamate, a serine at the n + 3 position can help create a CK2 substrate site if it is converted to phosphoserine by another protein kinase (Roach, 1991; Meggio and Pinna, 2003). The plastid transcription kinase PTK (cpCK2) is highly homologous to, and shares principal enzymatic properties with, the nucleocytosolic members of the CK2 family (Ogrzewalla *et al.*, 2002; Loschelder *et al.*, 2004), suggesting that prediction tools suitable for CK2 could also provide valid answers with regard to cpCK2 phosphorylation site(s) on AtSIG6.

**Table 1:** Prediction of putative casein kinase 2 (CK2) phosphorylation sites in AtSIG6

									Position in consensus motif							
Position	Aminoacid	NetPhos 2.0	NetPhosK 1.0	KinasePhos 2.0	disphos	gps	PREDPhospho	ScanSite	−2	−1	*	+1	+2	+3	+4	Homology
26	Serine	*				*			Y	S	S	P	S	S	V	
94	Serine	*	*	*	*	*	*		L	V	S	S	R	E	D	
95	Serine	*	*	*	*	*		*	V	S	S	R	E	D	E	
149	Serine	*							A	L	S	A	S	K	Q	
174	Serine	*		*	*	*			S	L	S	T	S	S	S	AtSIG2
176	Serine	*			*			*	S	T	S	S	S	M	S	
180	Serine	*			*	*	*	*	S	M	S	L	P	E	K	AtSIG5
206	Serine		*						P	K	S	N	D	V	D	
244	Threonine			*					P	E	T	K	Q	L	L	
249	Threonine		*						L	L	T	A	K	E	E	AtSIG1-5
282	Threonine		*						E	P	T	I	G	E	W	
306	Serine	*		*					G	R	S	S	R	E	K	
411	Serine	*		*		*			R	P	S	K	E	E	L	AtSIG4, 5
423	Threonine			*					V	S	T	E	K	L	D	
445	Serine			*		*			I	W	S	D	Q	D	T	
450	Threonine		*			*			D	T	T	F	Q	E	I	AtSIG4
458	Serine	*	*	*		*			P	D	S	G	I	E	T	AtSIG1, 3, 5
462	Threonine			*					I	E	T	P	T	M	S	AtSIG2, 3, 4
484	Serine	*		*		*			V	L	S	P	K	E	R	AtSIG4
504	Serine	*	*			*			Q	R	S	L	S	E	I	AtSIG1-5
CK2 consensus substrate site											S/T	x	x	E/D	x	

Prediction tools NetPhos 2.0 (Blom *et al.*, 1999), NetPhosK 1.0 (Blom *et al.*, 2004), KinasePhos 2.0 (Wong *et al.*, 2007), disphos (Diella *et al.*, 2004), gps (Xue *et al.*, 2005), PREDPhospho (Kim *et al.*, 2004) and ScanSite (Obenauer *et al.*, 2003) were used, leading to the detection of putative sites (+). Those selected for subsequent experiments are set in bold. The phosphoacceptor residue (*) and the acidic residue at the n + 3 position are marked in bold. Arabidopsis sigma factors showing regional similarity with AtSIG6 CK2 sites are indicated in the last column (Figure S2 for detailed positions). Bottom: consensus sequence of CK2 phosphorylation sites (Meggio and Pinna, 2003).

**Figure 1:**
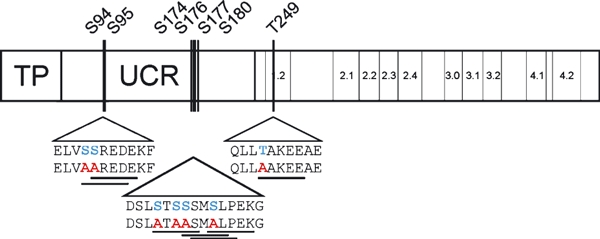
Positions of functionally tested cpCK2 phosphorylation sites on AtSIG6.Scheme showing the principal sigma factor architecture, with the conserved region containing subregions 1.2–4.2 on the right as well as the unconserved region (UCR) and the transit peptide sequence (TP) on the left. The sequences of the analysed substrate sites are enlarged below the triangles: wild type (upper row) and mutant (lower row). Original S or T residues that can function as phosphate acceptors are marked in blue, the exchanges are drawn in red and the entire cpCK2 site is underlined.

Indeed, using NetPhos 2.0 (Blom *et al.*, 1999), disphos 1.3 (Diella *et al.*, 2004), KinasePhos 2.0 (Wong *et al.*, 2007), NetPhosK 1.0 (Blom *et al.*, 2004), gps (Xue *et al.*, 2005), PREDPhospho (Kim *et al.*, 2004) and ScanSite (Obenauer *et al.*, 2003), a common picture emerges, suggesting a limited number of putative cpCK2 sites (Table 1). Based on their positions, these putative phosphorylation sites can be divided into ‘general’ and ‘unique’ sites, the former (those ranging from T244 to S504) being located within the CR, and the latter (from S26 to S206) being located within the UCR of the protein (Figure 1; Table 1).

**Figure 2:**
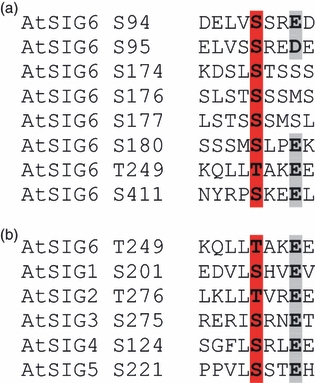
Alignment of analysed cpCK2 substrate motifs.The phosphoacceptor Ser or Thr residues are marked in red, and the acidic residues at n + 3 are marked in grey.(a) Multiple alignment of the eight cpCK2 substrate sites in AtSIG6 shown in Figure 1.(b) Alignment of the ‘general’ substrate site T249 from AtSIG6 with motifs from the conserved region of the other Arabidopsis sigma factors, AtSIG1– AtSIG5.

The ‘general’ site at T249 maps to the conserved sigma subregion 1.2 (Figure 1), which is known to be involved in core binding (Baldwin and Dombroski, 2001) and recognition control of the –10 promoter element (Zenkin *et al.*, 2007) in bacterial systems. Although only one of the seven prediction tools (NetPhosK 1.0; Table 1) gave an acceptably high score for this site, sequence alignments showed a putative CK2 site at equivalent positions in all Arabidopsis sigma factors (Figure 2b), as well as sigma factors from other plant species, e.g. maize ZmSIG6 (Beardslee *et al.*, 2002), *Chlamydomonas* RpoD (Carter *et al.*, 2004) and *Physcomitrella* PpSIG2 (Hara *et al.*, 2001) (Figure S1). A motif that would conform to the CK2 consensus site is noticeable even in bacterial sigma factors (Gruber and Gross, 2003), despite the lack of evidence for this kinase class in prokaryotes. Nevertheless, this ‘general’ site was included in subsequent analyses to allow for comparison with the ‘unique’ sites, i.e. potential CK2 substrate sites that are located within the UCR, and appear to be AtSIG6-specific.

Of the latter, those at S94 and S95 were detected by almost all prediction tools, and the assigned score values were in the top segment of all sites investigated (Figure 1; Table 1). Sequence alignment with the other Arabidopsis sigma factors did not reveal any appreciable similarity to AtSIG6 around S94/S95 (Figure S2). A somewhat similar situation applies to the region encompassing S174, S176 and S180, all of which are potential CK2 sites predicted by several (between three and five) programs. With one possible exception (residues reminiscent of S174 are located at positions T141 in AtSIG2), none of these sites is conserved in other Arabidopsis sigma factors (Figure S2).

### Phosphorylation control of recombinant AtSIG6 by PTK/cpCK2 *in vitro*

**Figure 3:**
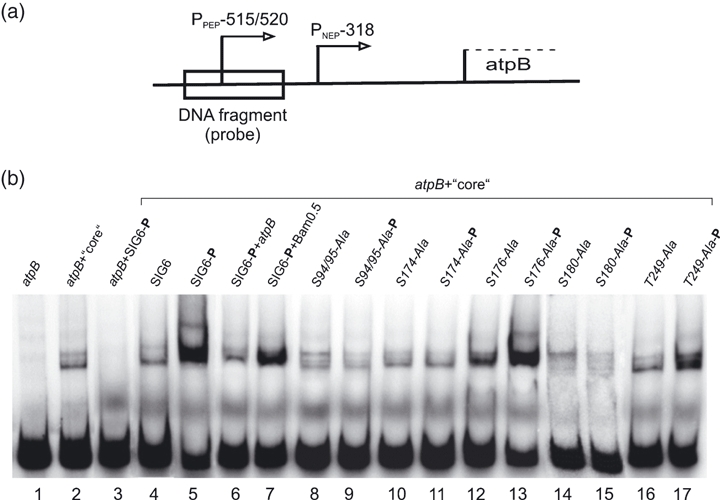
Phosphorylation-dependent DNA binding of recombinant AtSIG6 *in vitro*.(a) Map position of the DNA fragment used as a probe. The 424-bp fragment carries the *atpB* PEP-515/-520 promoter (Pfalz *et al.*, 2006) and a 154-bp section downstream of the transcription start site.(b) EMSA experiments using promoter fragment, *Escherichia coli* core polymerase and recombinant sigma proteins, with or without prior phosphorylation by recombinant cpCK2. The free radiolabelled DNA fragment migrates at the bottom (lane 1; *atpB*). Core polymerase alone (lane 2) but not AtSIG6 alone (lane 3) is able to bind to the promoter fragment. Complete reactions containing probe, core enzyme and either phosphorylated or mock-phosphorylated sigma proteins are shown in lanes 4–17 (*atpB* + core). Lanes 6 and 7 show competition with a twofold molar excess of unlabelled promoter fragment (*atpB*), but not with a promoter-less fragment Bam0.5 at 10-fold excess. Like AtSIG6 itself, none of the mutagenized derivatives show sigma activity in the unphosporylated form (lanes 8, 10, 12, 14 and 16).

To gain information on sigma factor activity in response to phosphorylation state, electrophoretic mobility shift assay (EMSA) DNA binding experiments were carried out using bacterially expressed AtSIG6 in combination with *E. coli* core RNA polymerase and a cloned DNA fragment that carries the Arabidopsis chloroplast *atpB* PEP promoter (Figure 3). The recombinant sigma factor was either used without (SIG6) or with prior phosphorylation by recombinant cpCK2 (SIG6-P).

Following incubation of either unphosphorylated (not shown) or phosphorylated AtSIG6 alone (lane 3) in the presence of the labelled probe, but without core enzyme, the only detectable signal is at the position of the free probe (bottom band). The virtual absence of any additional band with retarded mobility indicates that the factor lacks DNA binding activity on its own. *E. coli* core enzyme alone in the absence of AtSIG6 (lane 2) resulted in a small but significant portion of labelled material to a shifted position. The core enzyme preparation (Epicentre, http://www.epibio.com) did not contain detectable levels of bacterial sigma factor(s), and the shifted material was thus taken into account as the baseline in subsequent experiments.

The full system, i.e. the labelled probe in the presence of both AtSIG6 and the core enzyme (lane 4), gave shifted signals at the same position and strength as seen for the core alone (lane 2), suggesting that unphosphorylated AtSIG6 has little if any sigma activity in this assay. However, when the phosphorylated recombinant factor was used (SIG6-P, lane 5), this led to a considerable increase in intensity, along with an additional small shift in migration position of the DNA–protein complex. Carrying out the same reaction with an added twofold molar excess of unlabelled *atpB* promoter fragment as a competitor (lane 6), the intensity of shifted radioactive material was largely reduced. In contrast, a promoter-less fragment that was tested as a non-specific competitor did not negatively affect the binding signal (Bam0.5; lane 7) (Homann and Link, 2003). Together, this suggests that AtSIG6 indeed confers specific and efficient promoter binding to the polymerase, yet only in its phosphorylated form.

To investigate this further, we next constructed and tested mutant versions of the AtSIG6 protein that contained altered residues at putative cpCK2 substrate sites (Figure 2a). As is evident from Figure 3(b), neither the protein containing Ser → Ala exchanges at positions S94/95 (lanes 8 and 9), nor the one with an exchange at position S174 (lanes 10 and 11), revealed any significant SIG6-mediated DNA binding activity in the complete EMSA reaction, regardless of phosphorylation state. In contrast, the mutant version with a Ser → Ala exchange at S176 (lanes 12 and 13) showed a strong increase in DNA-binding activity upon phosphorylation, comparable with the effect observed for the non-mutagenized AtSIG6 protein (lanes 4 and 5). This suggests that S94/95 as well as S174, but not S176, are critical positions for phosphorylation-dependent activation of AtSIG6 *in vitro*.

A Thr → Ala exchange at the ‘general’ position T249 results in a mutant protein that is still active in its phosphorylated state (lanes 16 and 17), although perhaps to a somewhat lesser extent than the wild-type factor (lanes 4 and 5). This would argue against a significant regulatory effect of phosphorylation at this site in the *in vitro* system, at least for the *atpB* promoter.

### Construction and phenotypic analysis of Arabidopsis *AtSig6* mutant lines containing altered phosphorylation sites

**Figure 4:**
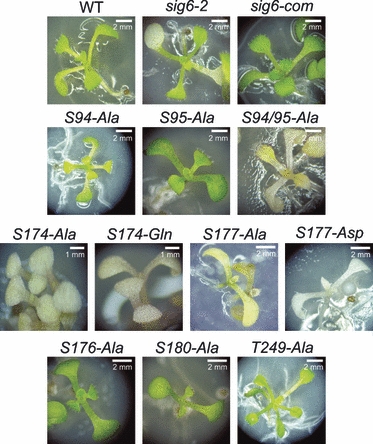
Phenotypic features of Arabidopsis *sig6* mutant lines carrying altered phosphorylation sites.Plates show 14-day-old plantlets representing the wild type (WT), the parental *sig6-2* knock-out line and the retransformed lines generated by cDNAs for either authentic AtSIG6 (*sig6-com*) or AtSIG6 derivatives, with exchange of residues 94, 95, 174, 176, 177, 180 or 249, as indicated. Several of the latter lines resemble the wild type in growth and pigmentation (*S94-Ala*, *S95-Ala*, *S176-Ala*, *S180-Ala* and *T249-Ala*), whereas others reveal a chlorophyll-deficient phenotype (*S94/95-Ala*, *S174-Ala*, *S174-Gln*, *S177-Ala* and *S177-Asp*) comparable with that of the parental *sig6-2* knock-out (*sig6-2*). S, serine site; T, threonine site.

**Figure 5:**
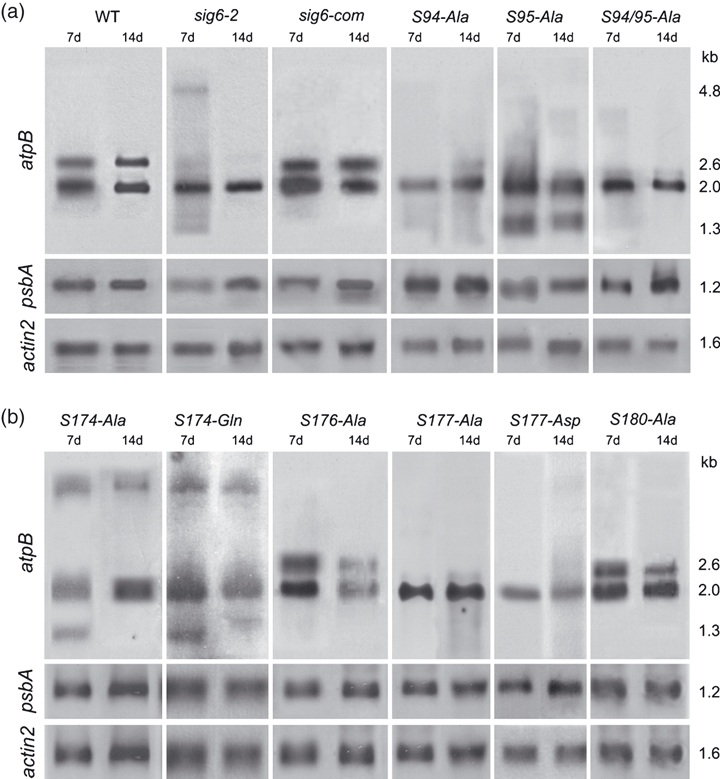
Site-directed phosphomutants are differentially affected in plastid gene expression. Northern blot analyses were carried out with total cellular RNA (1 μg each) from the wild type (WT), the *AtSig6* knock-out line (*sig6-2*) and the retransformed lines, including those containing fully functional AtSIG6 cDNA (*sig6-com*) and various point mutant derivatives. Hybridization was performed using DIG-labelled RNA probes for *atpB*, *psbA* and a nuclear control gene (*actin2*), with RNA from 7-day-old seedlings (7d) and 14-day-old plantlets (14d). Transcript sizes (kb) are indicated in the right margin.

Having located putative cpCK2 phosphorylation sites on AtSIG6 (Figures 1 and 2), and demonstrated phosphorylation-dependent activation of the factor *in vitro* (Figure 3b), we next asked which of these sites might be functionally relevant in Arabidopsis *in vivo*. To clarify this, we constructed full-length AtSIG6 cDNAs including the transit peptide region, either wildtype or mutant sequences, with amino acid exchanges at selected positions (Figures 1 and 2a). Following mobilization to a binary vector and retransformation of the Arabidopsis *sig6-2* knock-out, the progeny were analysed both for their visual (Figures 4 and S3) and molecular phenotypes (Figure 5).

To facilitate the identification of significant phenotypic traits, mutant lines were studied at two developmental stages, i.e. 7-day-old seedlings (Figure S3) and 14-day-old plantlets (Figure 4). They were compared with the wild type and the parental *sig6-2* knock-out line (Figures 4 and S3, first row), with the latter showing the previously described phenotype of white cotyledons but green true leaves (Loschelder *et al.*, 2006; Schweer *et al.*, 2006, 2009).

A number of phosphorylation site mutants that were tested exhibit the fully green wild-type phenotype: S94-Ala and S95-Ala (Figures 4 and S3, second row) as well as S176-Ala, S180-Ala and T249-Ala (fourth row). None of these lines showed gross deviation in growth parameters (size/shape) and pigmentation. However, in several other lines dramatic defects in pigmentation were found. These included the S94/95-Ala double exchange mutant (second row), as well as the mutant lines obtained after exchange at position S174 (S174-Ala and S174-Gln) or position S177 (S177-Ala and S177-Asp) (third row).

Within this chlorophyll-defective group of mutants, both the severity and the temporal mode of the pigment deficiency varied considerably. The most dramatic situation is found in the case of the S94/95 double mutant, which not only revealed white cotyledons (Figure S3) but also achlorophyllous primary leaves (Figure 4), leading to lethality at this stage. Not quite as extreme, but still more severe than the parental *sig6-2* knock-out, the S174 and S177 mutants develop white and yellowish cotyledons, respectively, and their first true leaves also remain pigment deficient. Although pigmentation gradually recovers to wild-type levels, both the S174 and S177 mutants remain compromised, as indicated by their delayed growth.

### Plastid gene expression in *AtSig6* phosphorylation site mutants

Northern blot hybridization has previously proved useful as a rapid diagnostic means to define SIG6-related organellar gene expression states in wild-type and *AtSig6* mutant lines (Loschelder *et al.*, 2006; Schweer *et al.*, 2006, 2009). Figure 5 shows a comparative analysis of phosphorylation site mutants following hybridization of total RNA samples (day 7 and 14) with either a chloroplast *atpB* or *psbA* probe, or with a nuclear *actin2* gene probe. Data obtained with the wild type, the parental *sig6-2* knock-out line, and the knock-out line retransformed and complemented with SIG6 cDNA were included as controls, to which the various phosporylation site mutants were compared.

In brief, with the *atpB* probe, the wild-type RNA sample reveals the two major *atpB/E* transcripts at 2.6 (SIG6/PEP-driven) and 2.0 kb (NEP-driven) (Schweer *et al.*, 2006). The knock-out mutant lacks the 2.6-kb transcript but shows the smaller 2.0-kb species and a transient 4.8-kb transcript. Following the retransformation of the *sig6-2* knock-out with SIG6 cDNA (*sig6-com*), the wild-type pattern is restored, i.e. both the 2.6- and 2.0-kb transcripts but not the 4.8-kb transcript are visible.

When RNA samples from the SIG6 phosphorylation site mutants were analysed, the hybridization patterns obtained were as follows: (i) The 2.0-kb (NEP) transcript is detectable in all lines; (ii) the larger 2.6-kb (SIG6/PEP) transcript is present at wild-type levels in both the S176 and the S180 seedlings, but is highly reduced or virtually absent in the other retransformed mutant lines; (iii) the 4.8-kb transcript is only detected in the S174 lines.

Based on these diagnostic transcript patterns, it seems that Ser174 is absolutely required for SIG6 activity in driving *atpB* gene expression *in vivo*. A noticeable, but less pronounced, effect is also obvious for Ser94 and Ser95, as well as for the ‘general’ site T249-Ala, whereas Ser176 and Ser180 do not seem to be required for activity.

Also shown in Figure 5 are the results of northern blot analyses with a *psbA* probe. The single 1.2-kb transcript is present in about equal relative quantities in wild-type RNA from 7-day-old seedlings and 14-day-old plantlets. In the *sig6-2* knock-out mutant the signal intensity is reduced only at the earlier stage, and its intensity is restored to wild-type levels following retransformation of the knock-out with SIG6 cDNA (Loschelder *et al.*, 2006). All phosphorylation site mutants show comparable transcript levels at both time points, i.e. they resemble the wild type rather than the parental knock-out with regard to *psbA* gene expression. Finally, the results obtained with an *actin2* gene probe establish that all mutant lines do not show significant deviation from the wild-type RNA expression levels of this nuclear control gene.

## Discussion

Here we have investigated putative cpCK2 phosphorylation sites in Arabidopsis sigma factor AtSIG6, and we have tested these sites for regulatory function. Together, the EMSA *in vitro* experiments (Figure 3) and the mutational analysis *in vivo* (Figures 4 and 5) support the notion that AtSIG6 is a cpCK2 substrate that responds to phosphorylation, resulting in altered DNA binding activity *in vitro* and changes in plastid gene expression patterns *in vivo*. Furthermore, based on the observation that *atpB* and *psbA* gene expression is differentially affected (Figure 5), the results obtained with the Arabidopsis *AtSig6* mutants suggest apparent promoter specificity of the phosphorylation control.

**Figure 6:**
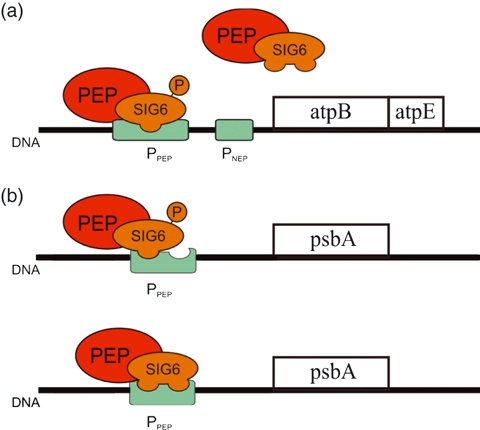
Model illustrating regulation by AtSIG6 phosphorylation at two different chloroplast promoters.(a) Transcription of the *atpB/E* operon is driven both from an NEP and a PEP promoter. The latter is recognized by the PEP/SIG6 complex, yet only if the sigma factor is phosphorylated at defined regulatory site(s).(b) In contrast, there is no apparent effect of SIG6 phosphorylation state on transcription at the *psbA* PEP promoter. The more complex architecture of this promoter (Figure S4 could be part of a scenario in which SIG6 is capable of binding at various phosphorylation sites, perhaps even further complicated by functional overlap with other sigma factors.Colour code: green, promoters; red, PEP; orange, AtSIG6.

As depicted in the model shown in Figure 6(a) for *atpB* transcription, AtSIG6 is able to confer productive binding to, and initiation from, the PEP promoter only in its phosphorylated state. The most critical phosphorylation sites seem to be those at S94/95 and S174, as inferred from the data indicating that mutational changes at these sites lead to dramatic alterations in phenotype (Figure 4) and plastid gene expression at the *atpB* PEP promoter *in vivo* (Figure 5). Interestingly, none of the analysed mutant lines show any appreciable effect on *psbA* gene expression *in vivo* (Figure 5). Furthermore, EMSA control experiments using the *psbA* promoter (Figure S5) did not reveal differences in binding activity of the phosphorylated versus unphosphorylated forms of AtSIG6. Together, this could mean that binding and initiation at the *psbA* promoter (Figure 6b) by AtSIG6 may be regulated differently compared with the *atpB* promoter (Figure 6a).

This is likely to reflect differences in promoter architecture, including the presence of the TATA-box-like element and extended –10 element (Browning and Busby, 2004) within the *psbA* but not *atpB* promoter (for a review, see e.g. Liere and Maliga, 2001; Swiatecka-Hagenbruch *et al.*, 2007). It could thus be envisaged that more than one choice exists for the efficient three-dimensional arrangement of the AtSIG6-PEP initiation complex at the *psbA* promoter. Furthermore, the *psbA* promoter is known to be recognized by more than one member of the sigma factor family (Hanaoka *et al.*, 2003; Privat *et al.*, 2003). Although *psbA* transcript levels are downregulated in *AtSig2*, *AtSig5* and *AtSig6* knock-out mutants, at least during certain developmental stages, the mRNA is never completely absent, which is consistent with the view that multiple sigma factors can initiate transcription from this promoter (Figure S4). In contrast, at the *atpB* promoter AtSIG6 does not seem to act in a redundant overlapping manner with other sigma factors (Schweer *et al.*, 2006), and thus mutational defects would be readily detectable in gene expression patterns from the *atpB/E* transcription unit (Schweer *et al.*, 2009).

Our present work establishes that only some of the putative cpCK2 phosphorylation sites on AtSIG6 are indeed utilized for regulation *in vitro* and/or *in vivo*. These include the two clusters of closely spaced sites S94/S95 and S174, S177 and S180.

The putative S94 and S95 sites each had high prediction scores with most of the programs used (Table 1). The positions n + 3 and n + 4, in the case of S94, and n + 2 through n + 4, in the case of S95, represent acidic residues in accordance with the CK2 consensus substrate site (Pinna, 1990). The exchange of Ser94 or Ser95 with alanine in each case affects the central phosphoacceptor residue of the consensus substrate site, which results in dramatic losses of the 2.6-kb *atpB* transcript originating from the *atpB* PEP promoter (Figure 5). Interestingly, however, both phosphorylation site mutants have a fully green phenotype resembling that of the wild type. Only if both Ser94 and Ser95 are mutated simultaneously (S94/S95 double mutant) is a chlorophyll-defective phenotype (Figure 4), as well as complete absence of the 2.6-kb *atpB* transcript (Figure 5), observed.

Consistent with these *in vivo* data, the EMSA experiments (Figure 3b) demonstrate that upon conversion of both Ser94 and Ser95 to Ala, the resulting mutant factor is no longer able to confer promoter-specific DNA binding in a phosphorylation-dependent manner *in vitro*. It is likely that Ser94 and Ser95 can both be used as phosphoacceptor residues in Arabidopsis, which may be an evolutionary safeguard to help warrant phosphorylation in this critical region of AtSIG6. Furthermore, it cannot be excluded that S94 and S95 are used alternatively during different developmental stages, and in different environmental situations. This idea is supported by the findings that the S94-Ala line showed highly diminished but still detectable levels of the 2.6-kb PEP transcript at 14 days, with a complete absence at 7 days, whereas the converse was true for S95-Ala (Figure 5).

The second region with clustered motifs for CK2 phosphorylation sites spans five serine residues between position 174 and 180 (Figure 1). Among these, only Ser174 and Ser177, but not Ser176 and Ser180, showed significant phenotypic effects *in vivo* upon conversion to other residues (Figures 4, 5 and S3). In addition, the Ser residues at 174 and 180, but not 176, appeared to be critical for phosphorylation-dependent DNA-binding activity *in vitro* (Figure 3b). It is notable that S174 is a ‘non-consensus’ CK2 substrate site because of the absence of an acidic (Asp or Glu) residue at position n + 3 (Pinna, 1990). However, this could be overcome by phosphorylation of the Ser at this position (Roach, 1991), which is a situation not rarely encountered in CK2 sites in other systems (Meggio and Pinna, 2003). Pre-phosphorylation of the n + 3 serine can be considered as a mechanism that ‘opens’ the CK2 site for phosphorylation in a temporal and/or spatial context of a kinase cascade. It should be noted, however, that a mimic phosphorylation experiment by Ser → Asp exchange at position S177 led to a chlorophyll-defective phenotype (Figure 4), and to a loss of the 2.6-kb *atpB* PEP transcript (Figure 5). Thus, the conclusion is that an ‘always on’ state at this position affects the SIG6 *in vivo* activity and the seedling phenotype in a negative, rather than positive, manner_._

How could pre-phosphorylation at the n + 3 residue of the S174 site be envisaged? In principle, cpCK2 itself could qualify as a ‘pathfinder’ kinase, taking advantage of the two closely-spaced serine residues Ser177 and Ser180. Phosphorylation at Ser180 (the n + 3 position for Ser177) would ‘open’ Ser177, which can then serve as the n + 3 position for Ser174. This explanation, which is solely based on our current knowledge of consensus CK2 substrate sites, is fully consistent with the data obtained in the *in vitro* DNA binding assays (Figure 3b). It is also consistent with most of the *in vivo* RNA data (Figure 5), except those for line S180-Ala. As the latter is defective at Ser180, cpCK2 should not be able to ‘open’ Ser177, or ultimately Ser174. Nevertheless, the S180-Ala line reveals the 2.6-kb PEP transcript, despite its defective Ser180 site, which could mean that Ser177 is pre-phosphorylated by an alternative kinase *in vivo*.

Screening for other potential kinases that might be able to recognize Ser177 suggested ribosomal S6 kinase (RSK; group 4.2.6 according to PlantsP, http://plantsp.genomics.purdue.edu/html/families.html) as a possible candidate. In recent localization analyses, a member of the RSK family, PK-like protein (At3g44610), was identified as a plastid kinase (Kleffmann *et al.*, 2004; Schliebner *et al.*, 2008). Another kinase with reasonable score is CK1, a nucleocytosolic enzyme with functions in intracellular protein targeting (Marin *et al.*, 2003). It is known that cytosolic phosphorylation can have an impact on chloroplast import (Martin *et al.*, 2006) and transcription (Christopher *et al.*, 1997). It would be interesting to investigate Arabidopsis kinase mutants to further test a regulatory role on sigma factor pre-phosphorylation.

PSIPRED (http://bioinf.cs.ucl.ac.uk/psipred; Jones, 1999) was used to distinguish between phosphorylation and alterations in secondary structure as a result of amino acid exchange. This tool suggested that there were no conformational differences in AtSIG6 compared with the site-directed mutant versions studied here. Furthermore, we experimentally addressed this question by the exchange of Ser for more than one alternative residue. For instance, as is evident from Figures 4 and S3 (third row), both Gln and Ala gave comparable results in the case of the S174 phosphorylation site.

The large-scale involvement of cpCK2 in generating the chloroplast phosphoproteome has recently been highlighted (Reiland *et al.*, 2009). Of the almost 200 detectable chloroplast phosphoproteins, many seem to reflect CK2 activity. These results underline a central role of cpCK2 in controlling the crosstalk between chloroplast gene expression and general metabolism. Other reports have previously pinpointed the role of protein kinase(s) in the regulation and maintenance of the chloroplast transcription apparatus (Kim *et al.*, 1999; Baena-Gonzalez *et al.*, 2001; Jeong *et al.*, 2004; Puthiyaveetil *et al.*, 2008; Steiner *et al.*, 2009). The critical role of nucleocytosolic CK2 in plant development and function has recently been established by using dominant-negative mutant lines (Moreno-Romero *et al.*, 2008). Chloroplast protein kinases with key functions in organellar signalling have been characterized using molecular genetic techniques (Depege *et al.*, 2003; Bonardi *et al.*, 2005; Vainonen *et al.*, 2005), whereas this is yet to be achieved for cpCK2. By addressing the question of how cpCK2 phosphorylation controls the activity of plastid sigma factors, our data reported here can help gain further insight into the role of this important signalling kinase in plants.

## Experimental procedures

### Plant growth conditions, harvesting and RNA extraction

*Arabidopsis thaliana* knock-out line *sig6-2* was obtained from the GABI-Kat mutant collection at the Max-Planck Institute fuer Zuechtungsforschung (GABI-Kat identifier 242G06; http://www.gabi-kat.de). It has a single T-DNA insertion in exon 5 of the *AtSig6* gene (Rosso *et al.*, 2003; Loschelder *et al.*, 2006). Wild-type, *sig6-2* and retransformed *sig6-2* seedlings (all *A. thaliana* ecotype Columbia) were grown on MS agar medium containing 0.4% (w/v) Gelrite and 1% (w/v) sucrose (Sigma-Aldrich, http://www.sigmaaldrich.com). Plates were maintained at 24°C under 8-h short-day conditions at a photofluence rate of 60 μmol m^−2^ sec^−1^. Cotyledons (day 7) and leaves (day 14) were collected, frozen and powdered in liquid nitrogen. Total RNA was prepared as decribed by Chomczynski and Sacchi (1987).

### Re-transformation of the *sig6-2* knock-out line

The full-length AtSIG6 cDNA including the transit peptide was mutagenized by using the QuikChange II site-directed mutagenesis kit (Stratagene, http://www.stratagene.com) and the oligonucleotides listed in Table S1. Products were cloned behind the CaMV 35S promoter of the binary vector pBINAR (Höfgen and Willmitzer, 1990). Each 35S promoter::cDNA construct was introduced into *Rhizobium radiobacter* (*Agrobacterium tumefaciens*) strain GV3101, and then transformed into the *sig6-2* line by floral dip (Clough and Bent, 1998). Complemented T_1_ plants were selected by kanamycin resistance, followed by Southern blot analysis and PCR with primers for the resistance gene (*npt1* and *npt2*) as well as those from within the *AtSig6* coding region (*UKSIG6-RP* and *UKSIG6-LP*) (Loschelder *et al.*, 2006). For each mutation, at least three independent lines were maintained and re-tested twice, except for the lethal double-mutant S94/95-Ala (six independent lines were tested once).

### Northern blot analysis

Gene-specific RNA probes (Loschelder *et al.*, 2006) were obtained by *in vitro* transcription of DNA regions cloned in pGEM-T Easy (Promega, http://www.promega.com). The linearized plasmids were transcribed by SP6 or T7 RNA polymerase in the presence of DIG-11-UTP (Roche, http://www.roche.com). Plant total RNA (1 μg) was gel-fractionated, transferred to positively charged nylon membrane (Roche) and hybridized with the DIG-labelled probe, followed by chemiluminescence detection (Schweer *et al.*, 2006).

### Recombinant proteins and phosphorylation

AtSIG6 cDNA or mutagenized derivatives thereof were amplified using the forward primer Sig6-TP (Table S1), which prevents the synthesis of the transit peptide region. PCR products were each cloned into pMAL-c2x (NEB, http://www.neb.com), and recombinant proteins were purified on amylose resin according to the pMAL manual. Purified proteins (15 μg) were phosphorylated in 50-μl reactions containing 20 mm Tris-HCl, pH 7.5, 50 mm KCl, 10 mm MgCl_2_, 0.1 mm ATP und 15 μg cpCK2 at 30°C for 30 min, as described by Ogrzewalla *et al.* (2002). Mock phosphorylation was carried out under identical conditions, without ATP. The recombinant kinase had less than 1/10th of the activity of a native commercial CK2 preparation (NEB), but revealed the typical CK2-type features, including ATP/GTP usage, acidic substrate preference and heparin sensitivity (Baginsky *et al.*, 1997, 1999; Ogrzewalla *et al.*, 2002). SIG6 and its mutant derivatives are cpCK2 substrates *in vitro* (H. Türkeri and G. Link, unpublished data).

### EMSA

EMSA reactions (Fried and Crothers, 1981; Garner and Revzin, 1981) containing 30 pmol sigma protein, 5.7 pmol *E. coli* core RNA polymerase (Epicentre, http://www.epicentre.com), 5 ng ^32^P-labelled double-stranded probe (Figure 3a) and 3 μg poly[d(I-C)] were incubated in 50 μl binding buffer at 25°C for 15 min (Homann and Link, 2003). The probe was prepared by PCR-based cloning of a 424-bp fragment (positions 54 534–54 958 on chloroplast DNA circle; AP000423) that carries the Arabidopsis *atpB* PEP promoter, followed by cloning into pGEM T-Easy (Promega). After cutting out the fragment with *Bam*HI/*Sal*I (Promega), it was electrophoretically purified, eluted, and end-labelled using [γ-^32^P]ATP and polynucleotide kinase (NEB). The same fragment, but unlabelled, was used as a specific competitor. Bam0.5, a 500-bp DNA fragment from within the *trnK* intron (Homann and Link, 2003), served as a non-specific competitor. Competitors were used at up to 20-fold molar excess, and were added prior to the labelled probe. DNA–protein complex formation was analysed in triplicate on native 4% (w/v) polyacrylamide gels (37.5:1 acrylamide:bisacrylamide) in TBE buffer (50 mm Tris, 45 mm boric acid, 0.5 mm EDTA, pH 8.3). After drying, gels were analysed using an FLA-3000 phosphoimager (Fuji, http://www.fujifilm.com).

## Supporting Information

**Figure S1**. Alignment of conserved sigma factor subregion 1.2 in various plant species, showing the distribution of putative CK2 substrate motifs.

**Figure S2**. Alignment of the six Arabidopsis sigma factors reveals the presence or absence of homologous phosphorylation sites in CR and UCR, respectively.

**Figure S3**. Phenotype of 7-day-old seedlings from Arabidopsis wild type and SIG6 mutant lines.

**Figure S4**. Differences in chloroplast *psbA* and *atpB* PEP promoter architecture.

**Figure S5**. EMSA with AtSIG6 and its mutant derivatives using the *psbA* promoter.

**Table S1**. Oligonucleotides used for site-directed mutagenesis and cloning of AtSIG6 cDNA constructs as well as the EMSA probe fragment carrying the *atpB* PEP promoter.

Please note: As a service to our authors and readers, this journal provides supporting information supplied by the authors. Such materials are peer-reviewed and may be re-organized for online delivery, but are not copy-edited or typeset. Technical support issues arising from supporting information (other than missing files) should be addressed to the authors.
