# Supplementary figures and images for: AtSIG6, a plastid sigma factor from Arabidopsis, reveals functional impact of cpCK2 phosphorylation
Source: Plant J. 2010 Apr;62(2):192–202. doi: 10.1111/j.1365-313X.2010.04138.x (PMC2988416; doi:10.1111/j.1365-313X.2010.04138.x)

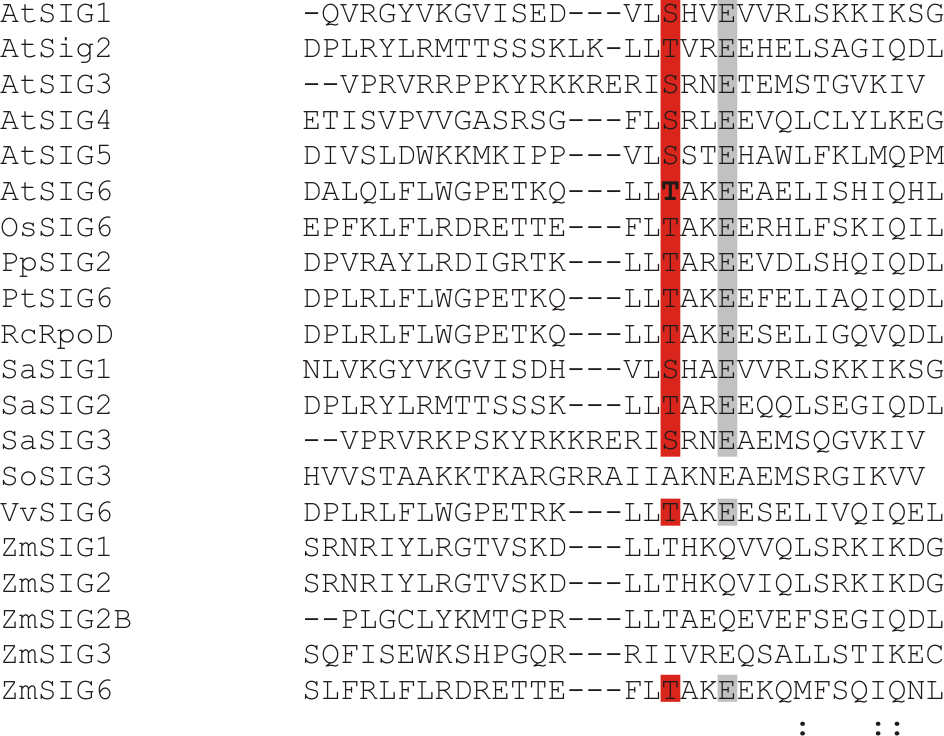

Supplement: Supplementary file 1 [file tpj0062-0192-SD1.tif]

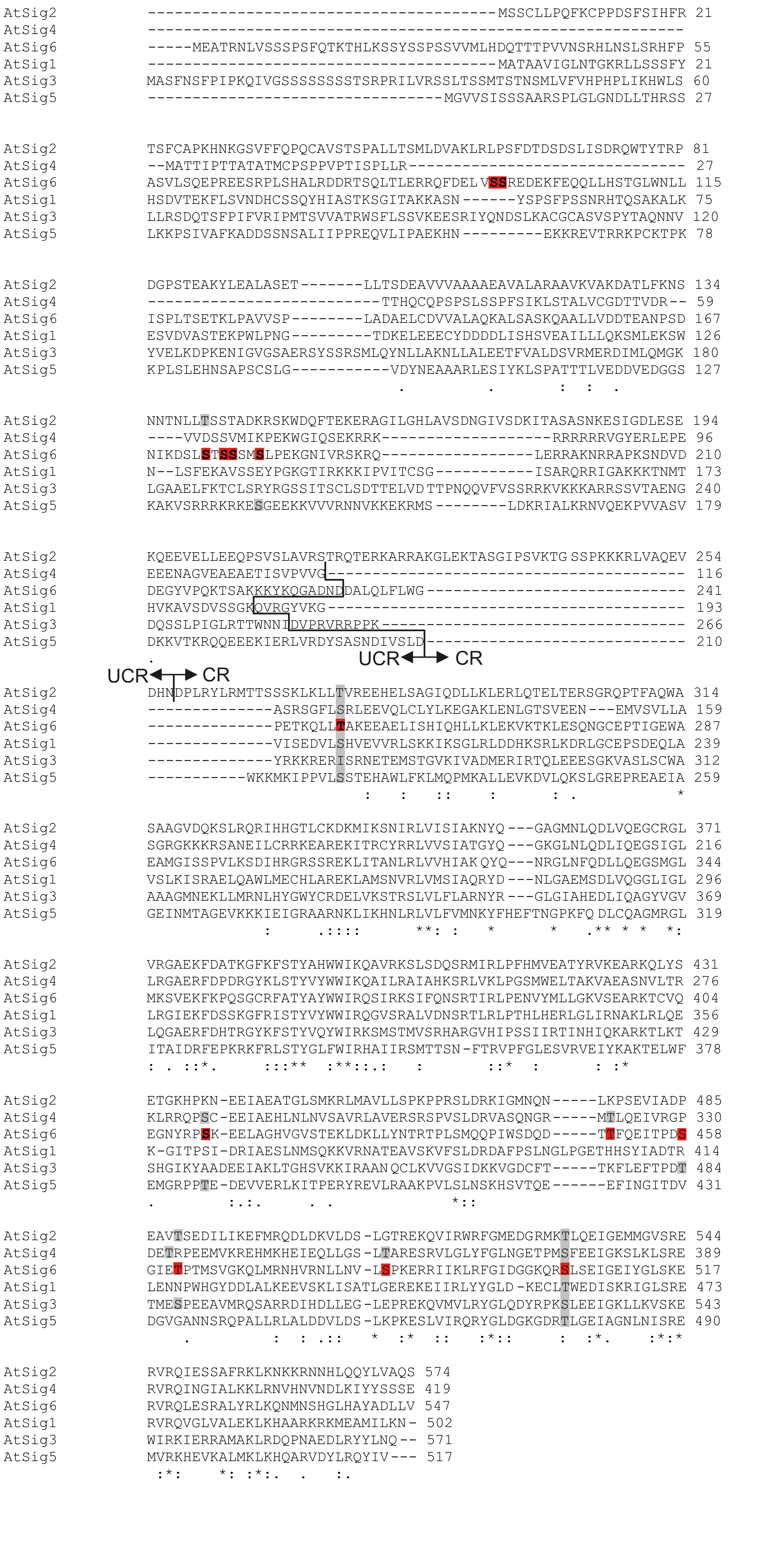

Supplement: Supplementary file 2 [file tpj0062-0192-SD2.tif]

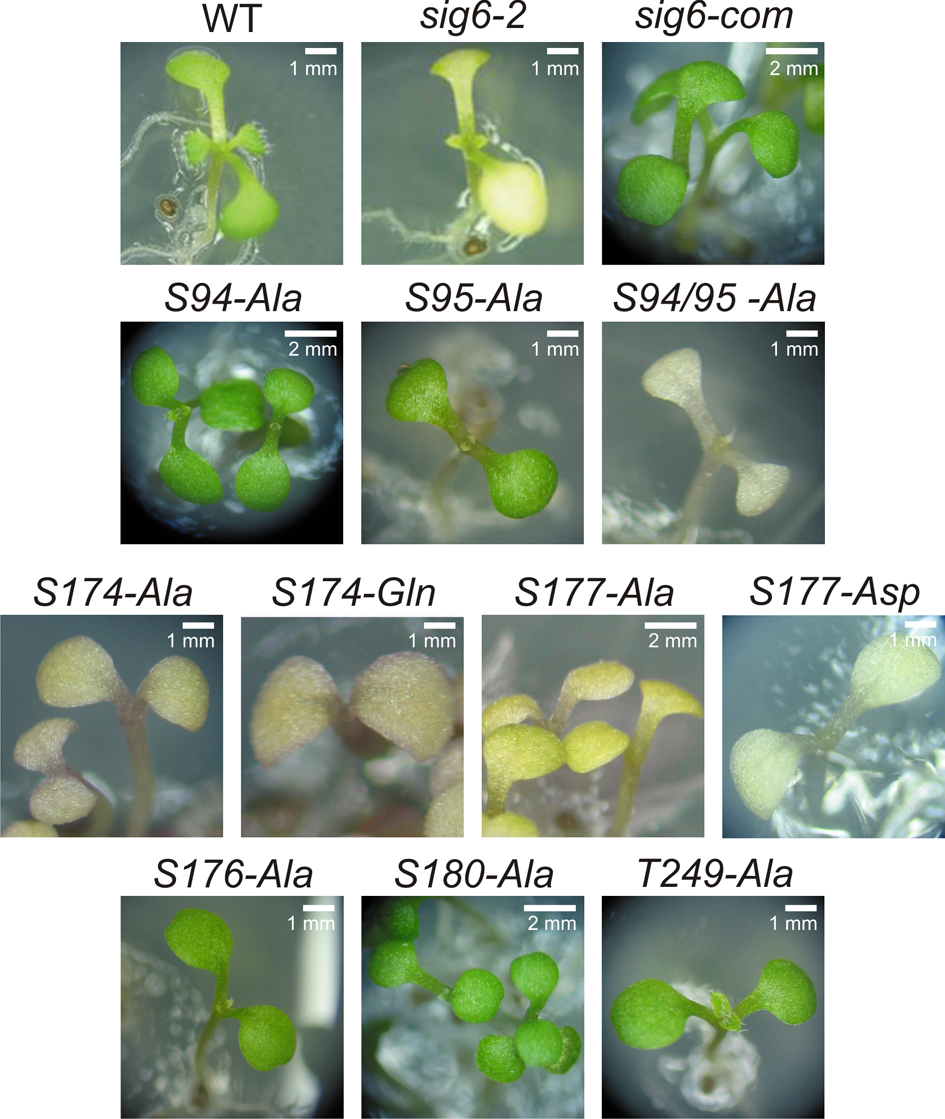

Supplement: Supplementary file 3 [file tpj0062-0192-SD3.tif]

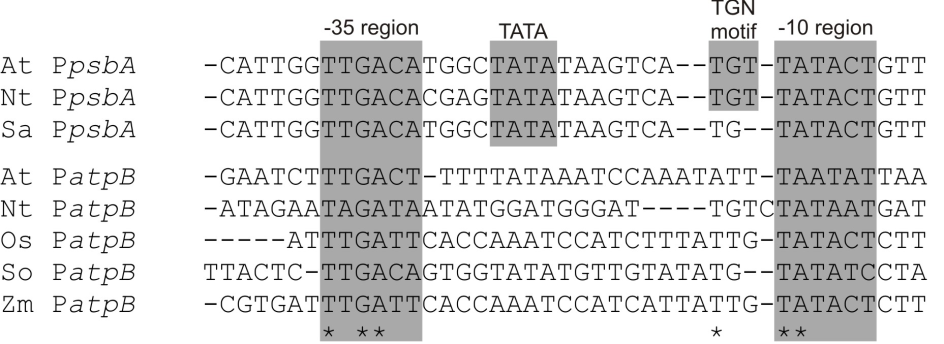

Supplement: Supplementary file 4 [file tpj0062-0192-SD4.tif]

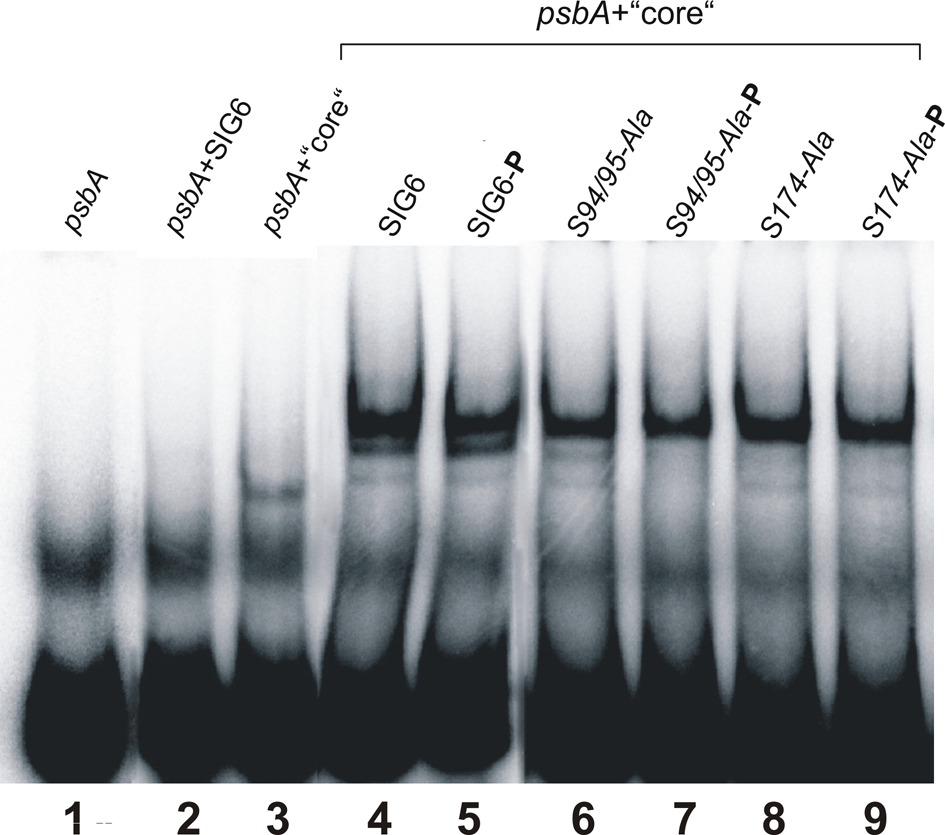

Supplement: Supplementary file 5 [file tpj0062-0192-SD5.tif]
