# Supplementary material for: AtSIG6, a plastid sigma factor from Arabidopsis, reveals functional impact of cpCK2 phosphorylation
Source: Plant J. 2010 Apr;62(2):192–202. doi: 10.1111/j.1365-313X.2010.04138.x (PMC2988416; doi:10.1111/j.1365-313X.2010.04138.x)
Supplement: Supplementary file 6 [file tpj0062-0192-SD6.doc]

**Table S1.** Oligonucleotides used for site-directed mutagenesis and cloning of AtSIG6 cDNA constructs as well as EMSA probe fragment carrying the *atpB* PEP promoter.

|  |  |
| --- | --- |
| Name | Sequence |
| S94-Ala | 5'-GGCAGTTTGATGAATTGGTTGCTTCTCCGAG-3'  3'-CTCGAGAAGCAACCAATTCATCAAACTGCC-5' |
|
| S95-Ala | 5'-GGCAGTTTGATGAATTGGTTTCAGCTCGAG-3'  3'-CTCGAGCTGAAACCAATTCATCAAACTGCC-5' |
|
| S94/95-Ala | 5'-GGCAGTTTGATGAATTGGTTGCTGCTCGAGAAGATGAG-3'  3'-CTCATCTTCTCGAGCAGCAACCAATTCATCAAACTGCC-5' |
|
| S174-Ala | 5'-GGATTCTCTCCAAACTAGCTCTTCTATGTCC-3'  3'-GGACATAGAAGAGCTAGTTTGGAGAGAATCC-5' |
|
| S174-Gln | 5'-GGATTCTCTCCAAACTAGCTCTTCTATGTCC-3'  3'-GGACATAGAAGAGCTAGTGTTGAGAGAATCC-5' |
|
| A176-Ala | 5'-GGATTCTCTCTCCACTCAATCTTCTATGTCC-3'  3'-GGACATAGAAGATTGAGTGGAGAGAGAATCG-5' |
|
| S177-Ala | 5'-CTCTCCACTAGCGCATCTATGTCCTTACCG-3'  3'-CGGTAAGGACATAGATGCGCTAGTGGAGAG-5' |
|
| S177-Asp | 5'-CTCTCCACTAGCGCATCTATGTCCTTACCG-3'  3'-CGGTAAGGACATAGATGCGCTAGTGGAGAG-5' |
|
| S180-Ala | 5'-CCACTAGCTCTTCTATGCAATTACCGGAAAAGGG-3'  3'-CCCTTTTCCGGTAATTGCATAGAAGAGCTAGTGG-5' |
|
| T249-Ala | 5'-GACAAAACAACTCTGGCTGCTAAAGAAGAAGC-3'  3'-GCTTCTTCTTTAGCAGCCAGAAGTTGTTTTGTC-5' |
|
| S411-Ala | 5'-GGGAATTACCGACCAGCTAAAGAGGAACTCGC-3'  3'-GCGAGTTCCTCTTTAGCTGGTCGGTAATTCCC-5' |
|
| Sig6-TP | 5'-GAATTCGGATCCGCGTCGGTTCTCTCACAGGAGCCA-3' |
| Sig6-for | 5'-ATGGAAGCTACGAGGAACTTGGTT-3' |
| Sig6-rev | 3'-CTAGACAAGCAAATCAGCATAAGC-5' |
| PatpB | 5'-TAGGCCAATACGCAGGGTAA-3'  3'-TTCAGGGAGGGATGTTCAGT-5' |
|
|  |  |
